# Maternal pre-pregnancy overweight/obesity and the risk of attention-deficit/hyperactivity disorder in offspring: a systematic review, meta-analysis and quasi-experimental family-based study

**DOI:** 10.1093/ije/dyaa040

**Published:** 2020-04-26

**Authors:** Lin Li, Tyra Lagerberg, Zheng Chang, Samuele Cortese, Mina A Rosenqvist, Catarina Almqvist, Brian M D’Onofrio, Tor-Arne Hegvik, Catharina Hartman, Qi Chen, Henrik Larsson

**Affiliations:** d1 School of Medical Sciences, Örebro University, Örebro, Sweden; d2 Department of Medical Epidemiology and Biostatistics, Karolinska Institutet, Stockholm, Sweden; d3 Department of Psychology, University of Southampton, Southampton, UK; d4 Pediatric Allergy and Pulmonology Unit at Astrid Lindgren Children’s Hospital, Karolinska University Hospital, Stockholm, Sweden; d5 Department of Psychological and Brain Sciences, Indiana University, Bloomington, IN, USA; d6 K.G. Jebsen Centre for Research on Neuropsychiatric Disorders, Department of Biomedicine, University of Bergen, Bergen, Norway; d7 University of Groningen, Groningen, Netherlands

**Keywords:** ADHD, obesity, meta-analysis, confounding, sibling comparison, cousin comparison

## Abstract

**Background:**

Previous studies are inconclusive concerning the association between maternal pre-pregnancy overweight/obesity and risk of attention-deficit/hyperactivity disorder (ADHD) in offspring. We therefore conducted a systematic review and meta-analysis to clarify this association. To address the variation in confounding adjustment between studies, especially inadequate adjustment of unmeasured familial confounding in most studies, we further performed cousin and sibling comparisons in a nationwide population-based cohort in Sweden.

**Methods:**

We searched PubMed, Embase and PsycINFO during 1975–2018. We used random-effects models to calculate pooled risk ratios (RRs) with 95% confidence interval. In the population-based study, Cox proportional hazard models were used to calculate the unadjusted hazard ratios (HRs) and HRs adjusted for all confounders identified in previous studies. Stratified Cox models were applied to data on full cousins and full siblings to further control for unmeasured familial confounding.

**Results:**

Eight cohorts with a total of 784 804 mother–child pairs were included in the meta-analysis. Maternal overweight [RR_overweight_ = 1.31 (1.25–1.38), *I*^2^ = 6.80%] and obesity [RR_obesity_ = 1.92 (1.84–2.00), *I*^2^ = 0.00%] were both associated with an increased risk of ADHD in offspring. In the population-based cohort of 971 501 individuals born between 1992 and 2004, unadjusted Cox models revealed similar associations [HR_overweight_ = 1.30 (1.28–1.34), HR_obesity_ = 1.92 (1.87–1.98)]. These associations gradually attenuated towards the null when adjusted for measured confounders [HR_overweight_ = 1.21 (1.19–1.25), HR_obesity_ = 1.60 (1.55–1.65)], unmeasured factors shared by cousins [HR_overweight_ = 1.10 (0.98–1.23), HR_obesity_ = 1.44 (1.22–1.70)] and unmeasured factors shared by siblings [HR_overweight_ = 1.01 (0.92–1.11), HR_obesity_ = 1.10 (0.94–1.27)].

**Conclusion:**

Pre-pregnancy overweight/obesity is associated with an increased risk of ADHD in offspring. The observed association is largely due to unmeasured familial confounding.

**Key Messages**

- Studies examining the effect of maternal pre-pregnancy overweight/obesity on risk of attention-deficit/hyperactivity disorder (ADHD) in offspring have only recently emerged and the findings are inconclusive.
- The causal status of the potential association between maternal pre-pregnancy overweight/obesity and risk of ADHD in offspring remains unclear.
- In a meta-analysis, we found that maternal pre-pregnancy overweight/obesity was associated with a higher risk of ADHD in offspring.
- Results from our family-based quasi-experimental study suggested that the observed association can be largely ascribed to unmeasured familial confounding, rather than a causal link.
- More studies with different methods and designs, in various populations or focusing on sever maternal obesity are still needed to replicate and build upon our findings.

## Introduction

Attention-deficit/hyperactivity disorder (ADHD) is a common and persistent neurodevelopmental disorder that is associated with adverse psychosocial, educational, occupational and health-related outcomes throughout life.^1^^,^^2^ ADHD affects approximately 6.5% of children and 2.5–3.4% of adults.^2^ The heritability of ADHD has repeatedly been found to be high, at 70–80%,^3^ but several environmental factors have been suggested to increase the risk of ADHD, e.g. prenatal and perinatal risks, dietary factors and psychosocial adversity.^4^^,^^5^ However, the mechanisms through which such risk factors influence ADHD remain unclear.

Maternal overweight and obesity prior to pregnancy are increasingly being recognized as potential modifiable risk factors for ADHD in offspring.^6^ Systematic reviews have suggested that maternal pre-pregnancy overweight/obesity may be associated with suboptimal neurodevelopment in offspring, including an increased risk for ADHD.^6–8^ Sanchez *et al*.^7^ conducted a meta-analysis on the association between maternal pre-pregnancy obesity and child neurodevelopmental outcomes and reported an overall effect of maternal pre-pregnancy overweight [OR_overweight_ = 1.30 (1.10–1.54), *I*^2^ = 52.97%] and obesity [OR_obesity_ = 1.62 (1.23–2.14), *I*^2^ = 70.15%] on ADHD in offspring but no sensitivity or subgroup analyses focused on ADHD specifically.

To date, the precise mechanisms underlying the association between maternal pre-pregnancy overweight/obesity and ADHD in offspring remain unclear. Some biological mechanisms have been proposed as mediators for a causal association, including fetal programming,^9^ placental and intrauterine environment alterations and inflammatory mechanisms.^10^ Alternatively, the association might be explained by unmeasured confounders. Indeed, recent register-based within-family studies^11^^,^^12^ have suggested that the associations of ADHD with high body mass index (BMI), including clinically diagnosed obesity, could be attributed to genetic factors shared by the two conditions. Additionally, a large genome-wide association study^13^ of clinically diagnosed ADHD reported a modest genetic correlation (r_g_) between ADHD and obesity-related phenotypes, including BMI (r_g_ = 0.26), waist-to-hip ratio (r_g_ = 0.30) and childhood obesity (r_g_ = 0.22). Unmeasured environmental confounders, such as lifestyle factors (e.g. dietary habits and physical activity), might also influence maternal overweight/obesity,^14^ as well as the risk of ADHD in offspring.^15^

Cross-generation observational studies evaluating the effect of maternal exposure on risk of ADHD in offspring also face the challenge of fully adjusting for genetic and environmental variables that are confounded with the hypothesized causal pathway. Previous systematic reviews and meta-analyses studies have discussed the limitations of trying to obtain a single answer using meta-analysis.^16^^,^^17^ These studies also provided examples on how to evaluate findings from meta-analyses by using population-based studies with fully adjusted measured confounding^17^ and suggested using genetically informative study designs (e.g. sibling or cousin comparisons) to help adjust for unmeasured genetic and environmental factors and to advance the understanding of the underlying processes through which early-life exposures influence later outcomes.^16^^,^^18^ However, only two previous studies^19^^,^^20^ have utilized sibling-comparison designs to address the role of unmeasured familial confounding in the context of maternal pre-pregnancy overweight/obesity and ADHD in offspring. Based on a nationwide population-based cohort study in Sweden including 272 790 full siblings born between 1992 and 2000, Chen *et al*.^19^ reported that the association between maternal pre-pregnancy overweight/obesity and ADHD in offspring was largely due to unmeasured familial factors. This finding was further replicated in a sample including 1958 siblings.^20^ However, these two studies were unable to fully examine the dose–response association between maternal pre-pregnancy overweight/obesity and risk of ADHD in offspring due to the limited numbers of included mothers with severe obesity (BMI ≥ 35). Indeed, the reduction of sample size and statistical power is an important limitation of the sibling-comparison design. Additionally, sibling comparisons rely on strong assumption (e.g. absence of carryover effects).^21^ Therefore, also the interpretation of these findings is unclear, given that women who change pre-pregnancy weight between pregnancies may be systematically different from women whose pre-pregnancy weight remains stable.^22–24^ Therefore, complementary designs, such as cousin comparisons, are needed to address these limitations.

In the current study, we first performed an updated systematic review and meta-analysis of the associations between maternal pre-pregnancy overweight/obesity and risk of ADHD in offspring with an extended included literature, detailed sub-analyses and detailed description for our confounding adjustment. To explain the findings of the meta-analysis and further evaluate the impact of confounding, a nationwide population-based cohort study was conducted by: (i) adjusting for all relevant measured covariates identified from Swedish medical registers, (ii) comparing first-born maternal full cousins and (iii) full siblings discordant with respect to maternal overweight/obesity to control for shared familial factors in extended families and nuclear families, respectively.

## Methods

### Systematic review and meta-analysis

We applied the standard methodological guidelines of the PRISMA (the Preferred Reporting Items for Systematic Reviews and Meta-Analysis) statement^25^ and registered our systematic review and meta-analysis on PROSPERO (International prospective register of systematic reviews) (CRD42018092267).

#### Search strategy and selection of studies

We systematically searched PubMed, Embase and PsycINFO using a pre-specified search strategy to identify all pertinent studies on humans published from 1 January 1975 to 31 December 2018, evaluating the association between maternal overweight or obesity and risk of ADHD in offspring. Detailed information on the search terms and syntax for each database are reported in Supplementary Table 1, available as Supplementary data at *IJE* online. No restrictions were imposed on language and date of publication. References of selected papers were hand searched by two authors (L.L. and T.L.) to retrieve any possible additional pertinent publication that could have been missed with the electronic search.

Published studies were included according to the following inclusion criteria: (i) case–control and cohort studies; (ii) offspring with ADHD defined with any of the following: DSM (Diagnostic and Statistical Manual of Mental Disorders) criteria (III, III-R, IV, IV-TR or 5), hyperkinetic disorder according to ICD-9 or ICD-10, ADHD-medication prescriptions as a proxy to diagnosis, physician diagnosis of ADHD, ADHD symptoms based on value above cut-off on a validated self-reported ADHD questionnaire, ADHD diagnosed via a structured psychiatric interview or positive answer by parents to the question ‘Has the child ever been told it has ADHD by a doctor?’ or similar ones; (iii) BMI calculated from either directly measured or self-reported body weight and height; (iv) studies reporting results as risk ratio (RR), hazard ratio (HR) or odds ratio (OR) with its corresponding 95% confidence interval (CI) or sufficient data (e.g. sample size, prevalence of ADHD, overweight and obesity) to calculate them. When needed, we contacted the corresponding author to acquire unpublished data to calculate the related effect size. When multiple reports containing overlapping participants were available, the article with the largest number of subjects and most applicable information was preferred.

#### Data extraction

The following data were extracted from each study retained for the qualitative synthesis: name of the first author, publication year, study location, number of participants, definition of exposure (maternal pre-pregnancy overweight or obesity), definition of outcome (ADHD), covariates and how these were handled, crude and adjusted effect size (OR/RR/HR/β) with 95% CIs.

#### Assessment of study quality

The Newcastle-Ottawa Scale (NOS), a validated tool for assessing the quality of observational studies, was used to assess possible bias in the included studies.^26^ The following three categories were evaluated with a maximum score of 9: selection (definition/representativeness of exposed subjects, selection of non-exposed subjects), comparability (controls or adjustment for confounding factors) and outcome (assessment of outcome, adequate non-response rate or follow-up time). Authors L.L. and T.L. independently graded all included studies using the NOS criteria and the discrepancies were solved by consensus.

#### Statistical analysis

The characteristics of the included studies and the heterogeneity in confounding adjustment strategies (i.e. various confounding adjustment strategies adopted by the available studies) were described in detail. ORs from logistic regression and HRs from Cox regression were combined because they closely approximate each other.^16^^,^^27^^,^^28^ The ORs were considered equivalent to RRs given the low prevalence of ADHD diagnosis.^29^ To be as inclusive as possible, we chose teacher-rated inattention symptoms as the main outcome in the studies with multiple definitions of ADHD. Fewer studies presented covariate-adjusted effect estimates for obesity, so crude RRs were included in the primary analyses, whereas adjusted RRs and 95% CIs were obtained for sensitivity analyses. A leave-one-out analysis was also conducted to assess whether a single study markedly affected the overall findings.

The following subgroup analyses were conducted: (i) including only studies with an ADHD diagnosis based on DSM (III, III-R, IV, IV-TR or 5) or ICD-10 or previous versions; (ii) analysing ADHD assessed from rating scales by parents, teachers and self-ratings and diagnostic criteria separately; (iii) analysing studies with self-reported vs measured BMI/overweight/obesity separately; (iv) analysing studies with pre-pregnancy and early-pregnancy BMI/overweight/obesity separately; (v) removing studies based on Swedish samples (to avoid any concern about possible overlap with the empirical study presented in this paper); (vi) analysing outcomes of overweight and different levels of obesity (obesity class I, II and III) separately.

Pooled-effect estimates were calculated using random-effects models to take into account heterogeneity between studies and the results were summarized in forest plots. Heterogeneity among studies was assessed by the Cochran Q test and *I*^2^ statistic (level of significance *P* < 0.10 and *I*^2^ > 70%, respectively). The presence of publication bias was first assessed through visual inspection of funnel-plot symmetry assessed and then assessed quantitatively with the Begg’s test and Egger’s test. All statistical analyses were conducted using Stata, version 15.1 (Stata Corp, College Station, TX, USA).

### Nationwide population-based cohort study

The nationwide population-based cohort study was approved by the regional ethical review board in Stockholm, Sweden. The requirement for informed consent was waived because the data were pseudonymized from population-based registers.

#### Data sources

With individual-specific personal identification numbers, we linked the following seven Swedish registers: (i) the Medical Birth Register (MBR) provided data on more than 95% of pregnancies in Sweden since 1973^30^; (ii) the National Patient Register (NPR) contained data on inpatient psychiatric care since 1973 (ICD-9 to ICD-10) and outpatient psychiatric care since 2001 (ICD-10)^31^; (iii) the Multi-Generation Register provided information on biological relationships for all residents in Sweden since 1932; (iv) the Prescribed Drug Register (PDR) included detailed information on drug identity [Anatomical Therapeutic Chemical (ATC) code] and dates of all registered prescriptions for all individuals residing in Sweden since 1 July 2005^32^; (v) the Swedish Register of Education provided data on highest education level through 2008; (vi) the Cause of Death Register provided detailed information on all registered deaths since 1958; (vii) the Migration Register included information on all migrations in or out of Sweden since 1969.

A total of 1 232 207 live-born individuals in Sweden were identified from the MBR between 1992 and 2004. We excluded those who had severe congenital malformations (*N* = 45 533), died (*N* = 3437) or emigrated (*N* = 21 715) before 3 years of age, lacked mother’s identification number (*N* = 382), received an ADHD diagnosis before 3 years of age (*N* = 76) or lacked information on maternal BMI (*N* = 189 563), resulting in 971 501 individuals as the final study population. We further identified 463 474 full biological siblings nested within 216 084 families and 155 841 first-born maternal full cousins nested within 74 057 extended families from the entire study population. All individuals were followed from the third birthday until a diagnosis of ADHD, death, emigration or 31 December 2013, whichever occurred first.

#### Exposure definition

Data on self-reported height and measured weight in light indoor clothing without shoes at the first prenatal visit (within the first 14 gestational weeks for 90% of pregnant women) were obtained from the MBR. Maternal BMI during early pregnancy (as a proxy of pre-pregnancy BMI) was calculated from weight in kilograms divided by height in metres-squared and classified into underweight (BMI < 18.5), normal weight (18.5 ≤ BMI < 25.0), overweight (25.0 ≤ BMI < 30.0), obesity class I (30.0 ≤ BMI < 35.0), obesity class II (35.0 ≤ BMI < 40.0) or obesity class III (BMI ≥ 40.0), according to the World Health Organization guidelines.^33^ In line with previous studies,^19^^,^^34–38^ we also identified an obesity group with all obesity classes combined (BMI ≥ 30). In addition, BMI was treated as a continuous exposure in some sensitivity analyses.

#### Outcome definition

Outcome was defined as time since the third birthday to first ever ADHD diagnosis or prescription of ADHD medication. Information on date of ADHD diagnosis was retrieved from the NPR, based on ICD codes (ICD-9: 314; ICD-10: F90). Information on date of ADHD-medication prescription was extracted from the PDR according to ATC codes (ATC: N06BA04, N06BA01, N06BA02 and N06BA09).

#### Covariates

**Figure 1:**
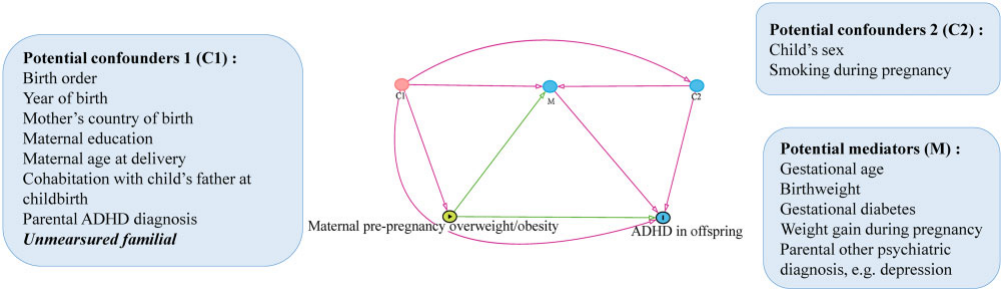
Casual diagram representing the potential pathways of the association between maternal pre-pregnancy overweight/obesity and risk of ADHD in offspring. C1: the potential common causes of maternal pre-pregnancy overweight/obesity and ADHD in offspring; M: the potential mediators on the pathway from maternal pre-pregnancy overweight/obesity to ADHD in offspring; C2: the potential common causes of mediators of the studies association and ADHD in offspring. ADHD, attention-deficit/hyperactivity disorder.

We constructed a directed acyclic graph (DAG),^39^ based on covariates used in previous studies and available data in the Swedish national registers, for covariate selection (Figure 1). In the current study, the selected covariates (potential confounders) included offspring sex, birth order (first, second, third or fourth) and year of birth (1992–1995, 1996–1999 and 2000–2004); mother’s country of birth (Sweden, other Scandinavian country or other); maternal education (≤9 years, 10–12 years or postgraduate education); maternal age at delivery (≤19, 20–24, 25–29, 30–34 or ≥35 years); smoking during pregnancy (0, 1–9 or ≥10 cigarettes per day); and cohabitation with child’s father at childbirth (yes or no). Information on parental ADHD was not available but shared by full siblings and thus implicitly adjusted by sibling-comparison design.

#### Statistical analyses

We used Cox proportional-hazards models to estimate the association between maternal overweight and obesity and risk of ADHD in offspring at the entire population level. Maternal overweight, obesity and obesity class I–III were all compared with normal weight. In accordance with the meta-analysis part, underweight women were not included in the analyses. The Cox models were adjusted for all measured confounders mentioned above. The results are presented as HRs with 95% CIs based on robust standard errors.

To explore the effects of unmeasured shared familial confounding on the observed association between maternal pre-pregnancy overweight and obesity and ADHD in offspring, stratified Cox proportional-hazards models were used for cousin and sibling comparisons, with each set of maternal full cousins and full siblings representing separate strata. A total of 24 521 extended families and 31 906 nuclear families contained first-born maternal full cousins and siblings discordantly exposed to maternal pre-pregnancy-weight status (normal-overweight/obesity and overweight/obesity-normal). The cousin-comparison models were implicitly adjusted for all unmeasured factors shared by cousins within each extended family (e.g. 12.5% shared genetic factors, racial and ethnic factors) and all measured birth-specific covariates as in the models at the population level, because all these measured covariates show variation within cousins. The sibling comparisons were implicitly adjusted for all factors shared by siblings within each nuclear family (e.g. 50% shared genetic factors, racial and ethnic factors, lifestyle factors), including maternal factors (birth country, highest education level); thus, only non-maternal birth-specific covariates were controlled in the sibling comparisons (offspring sex, birth order, year of birth, maternal age at delivery, smoking during pregnancy and cohabitation with child’s father at childbirth). Finally, continuous BMI was then used as exposure to examine the robustness of all above results.

We performed three sensitivity analyses to examine the robustness of our results. First, the included families differed in family size (two to eight siblings per family), but most of the families (86.79%) contributed with two siblings. In addition, later-born offspring were more often exposed to overweight or obesity. Therefore, we identified a sub-sample (*N* = 432 168) including only first- and second-born siblings from each family for sensitivity analysis. Second, using BMI as a continuous variable, we conducted a bidirectional case-crossover analysis by dividing participants with different weight patterns between pregnancies and repeated the main analyses. Hence, we could explore the potential influence of changing weight status and carryover effects (e.g. the exposure during first pregnancy may affect the outcomes during the second pregnancy) from one pregnancy to the next caused by different types of between-pregnancy variation in BMI (Normal-Normal, Normal-Overweight/Obesity, Overweight/Obesity-Normal and Overweight/Obesity-Overweight/Obesity). Finally, as suggested in a previous review,^40^ bariatric surgery for the severely obese has been consistently shown to lead to long-term weight loss and dramatic improvement in medical comorbidity (e.g. metabolic syndrome). Moreover, previous research^41^^,^^42^ showed improvement of cognitive functions and some ADHD symptoms after surgery. Together, this may indicate that bariatric surgery could confound the link between maternal pre-pregnancy obesity and risk of ADHD in offspring. Thus, to rule out potential bias by bariatric surgery, we excluded those whose mother had bariatric surgeries prior to any delivery (*N* = 14 028) and repeated our main analyses. Individuals who had undergone bariatric surgeries were identified from the NPR by using a Swedish adaption of the Classification of Surgical Procedures (NOMESKO) codes: 4750–4754, 4759, JDF00, JDF01, JDF10, JDF11, JDF20, JDF21, JDF32, JDF96, JDF97, JDF98, JFD00, JFD03, JFD04, JFD10, JFD13, JFD20, JFD23, JFD96.

All statistical analyses were conducted in SAS version 9.3 (SAS Institute, Cary, NC, USA).

## Results

### Meta-analysis

#### Study characteristics

**Figure 2:**
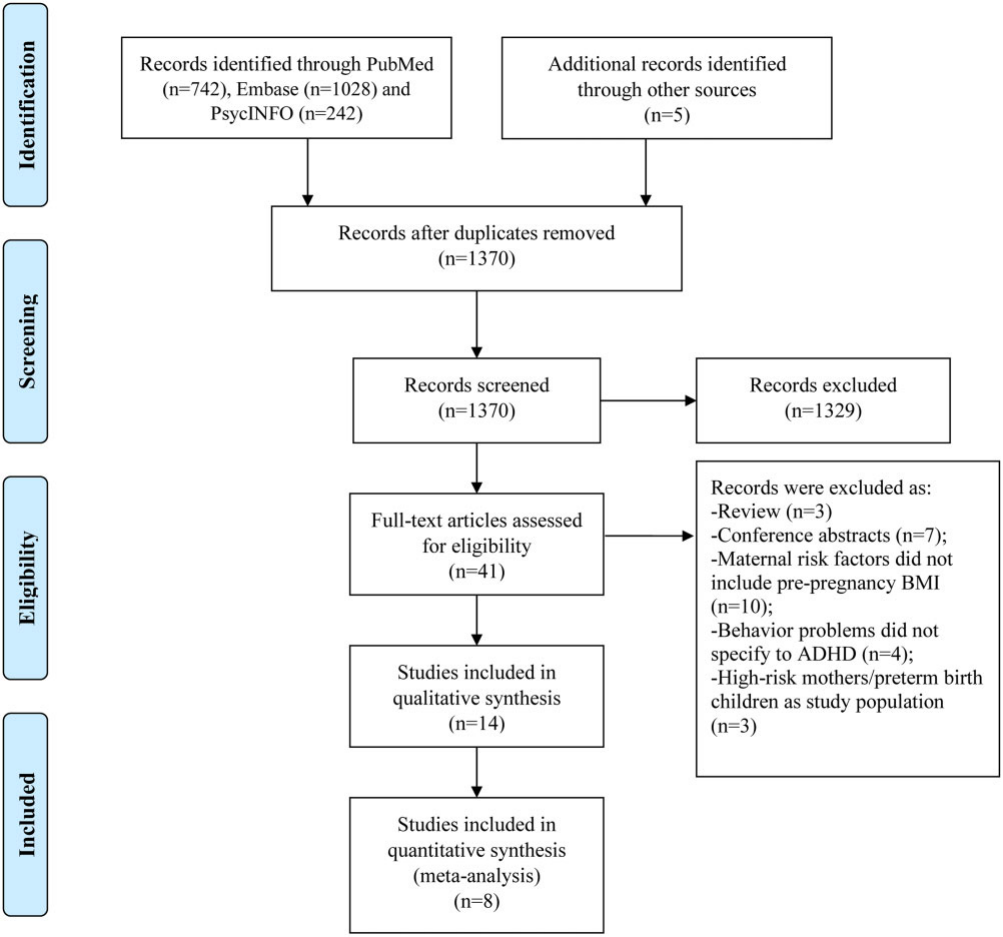
PRISMA flow diagram for inclusion of the studies examining the association between maternal overweight or obesity during pregnancy and ADHD risk in offspring. PRISMA, Preferred Reporting Items for Systematic Review and Meta-Analyses; ADHD, attention-deficit/hyperactivity disorder.

A total of 784 804 mother–child pairs from eight pertinent cohort studies^19^^,^^20^^,^^34^^,^^35^^,^^43–46^ were included in the meta-analysis (Figure 2). Another 41 825 pairs from six studies were only included in the qualitative synthesis because of limited information for effect size calculation,^36^^,^^37^^,^^47^^,^^48^ different definitions of exposure^49^ or overlapping study populations.^38^Table 1 shows the demographic and statistical details of the 14 studies published between 2008 and 2017 included in the systematic review. The size of the cohorts ranged from 112 to 673 632. For those included in the meta-analysis, overweight and obesity was the most frequent measure of exposure whereas four of the studies further divided obesity into Obesity Class I, II and III (or II/III). ADHD in offspring was assessed by a mother-reported previous ADHD diagnosis, clinical diagnosis from national patient registers or teacher/mother-reported ADHD symptoms based on DSM-IV, the Child Behavior Checklist (CBCL) and the Strengths and Difficulties Questionnaire (SDQ). For crude and fully adjusted effect size, maternal overweight, obesity and obesity class I–III were all compared with normal weight (18.5 ≤ BMI < 25.0).

**Table 1.** Overview of cohort studies included in the systematic review

First author (year)	Country	Sample (pairs)	Exposure				ADHD				Crude effect size (95% CI)		Fully adjusted effect size (95% CI )		Estimates	NOS
			Definition	Assessment	Time at assessment	Prevalence/ mean ± SD	Sources	Assessment	Age at assessment	Prevalence/ mean ± SD	Overweight	Obesity	Overweight	Obesity		
1) Rodriguez A (2008)	Sweden Denmark Finland	14 519	Overweight Obesity	Medical records	Around gestational week 10	Overweight: 9.9% Obesity: 1.8%	Teachers	SDQ (Swe/DK); RB2 (Fin)	7–12	8.52%/ 0.96 ± 1.55	1.24 (0.99–1.55)	1.98 (1.26–3.10)	1.37 (1.07–1.75)	1.89 (1.13–3.15)	OR	8
2) Rodriguez A^a^ (2010)	Sweden	1741	Overweight Obesity	Swedish Medical Birth Register	–	Overweight: 26.3% Obesity: 9.5%	Mothers and teachers	DSM-IV	5	2.5%	1.92 (1.21–3.05)	2.05 (1.06–3.95)	2.00 (1.20–3.35)	2.09 (1.19–4.82)	OR	8
3) Brion MJ^b^ (2011)	UK Netherlands	4873/ 3922	BMI > 25	Self-reported	Around gestational week 12	–	Mothers and teachers	SDQ	4/8	Median: 4	–	–	OR_4-year-old_ = 0.93 (0.82–1.05) OR _8-year-old_ = 1.06 (0.89–1.27)		OR	7
4) Buss C^b^ (2012)	USA	174	Overweight Obesity	Medical records	Pre-pregnancy	25.5 ± 5.9	Mothers	CBCL	7	0.45 ± 0.41	–	–	β = 0.18 *P* = 0.03		β	6
5) Hinkle SN (2013)	USA	5200	Overweight Obesity IObesity II/III	Self-reported	Pre-pregnancy	Overweight: 25.0% Obesity I: 8.6%Obesity II/III: 6.2%	Parent /primary caregiver	Previous diagnosis of ADHD	2–5	2.9%	0.80 (0.48–1.31)	1.64 (0.96–2.81)	0.68 (0.38–1.22)	1.80 (1.01–3.18)	RR	7
6) Chen Q (2014)	Sweden	673 632	Overweight Obesity	Self-reported	Around gestational week 10	Overweight: 22.1% Obesity: 7.9%	Registers	ICD-9/ ICD-10/ DSM-IV/ADHD medication	–	2.6%	1.31 (1.27–1.36)	1.95 (1.86–2.04)	1.23 (1.18–1.27)	1.64 (1.57–1.73)	HR	9
7) Van Mil^b^ (2014)	Netherlands	6015	BMI	Self-reported	Early pregnancy	24.6 ± 4.3	Parents	CBCL	6	–	β = 0.09 (0.04–0.14)		β = 0.04 (–0.01–0.10)		β	7
8) Jo H (2015)	USA	1311	Overweight Obesity IObesity II/III	Self-reported	Pre-pregnancy	Overweight: 25.4% Obesity I: 12.6% Obesity II/III: 12.2%	Mothers	Previous diagnosis of ADHD	6	3.1%	1.83 (0.81–4.12)	2.36 (1.11–5.03)	1.83 (0.76–4.39)	–	OR	6
9) Andersen CH (2017)	Denmark	81 892	Overweight Obesity IObesity II/III	Self-reported	Around gestational week 16	Overweight: 20% Obesity I: 7%Obesity II/III: 2%	Registers	ICD-9/10	Average 13.3	3.0%	1.34 (1.22–1.48)	1.72 (1.52–1.95)	1.28 (1.15–1.41)	–	HR	8
10) Musser ED (2017)	USA	4682	Overweight Obesity IObesity IIObesity III	–	Pre-pregnancy	27.6 ± 6.7	Registers	ICD-9/10	5–12	4%	1.33 (0.90–1.95)	1.93 (1.36–2.74)	1.54 (1.05–2.27)	–	OR	9
11) Casas M^a^ (2017)	Spain	1827	Overweight Obesity	Self-reported	Around gestational week 13.9	Overweight: 19% Obesity: 8%	Teachers	DSM-IV	5	–	1.01 (0.18–5.33)	1.55 (0.17–13.74)	1.05 (0.55–1.99)	1.40 (0.44–4.53)	IRR	8
12) Daraki V^b^ (2017)	Greece	581	Overweight Obesity	Self-reported	Pre-pregnancy	Overweight: 22% Obesity: 13%	Mothers	SDQ	4	–	–	–	β = –0.69 (–3.03–1.64)	β = 4.28 (1.20–7.36)	β	7
13) Mina TH^b^ (2017)	UK	112	Obesity III	Measured by midwives	Early pregnancy	Obesity III: 44.6%	Mothers	CBCL	4	–	–	–	β = 0.74 (0.25–1.22)		β	8
14) Mikkelsen SH^b^ (2017)	Denmark	32 163	Overweight Obesity	Self-reported	Pre-pregnancy	Overweight: 19% Obesity: 7%	Mothers	SDQ	7	4.9%	–	–	1.25 (1.10–1.42)	1.45 (1.23–1.73)	OR	9

a Provided effect size for different ADHD syndromes with different assessments; only teacher-reported attention-deficit symptoms were included.

b Not included in the meta-analysis.

ADHD, attention-deficit/hyperactivity disorder; SD, standard deviation; CI, confidence interval; OR, odds ratio; HR, hazard ratio; RR, risk ratios; NOS, the Newcastle-Ottawa Scale; BMI, body mass index; CBCL, Child Behavior Checklist; SDQ, the Strengths and Difficulties Questionnaire; ICD, International Classification of Diseases; DSM-IV, Diagnostic and Statistical Manual of Mental Disorders.

The quality scores based on the NOS ranged from 6 to 9, suggesting an overall high quality of the included studies. As shown in Table 2, the number of stars represented the score of each item. Most studies used well-defined exposures and outcomes, with strict selection criteria. However, some included studies with one star in ‘Comparability’ did not consider familial factors as potential confounders (e.g. genetic factors, paternal characteristics). The adjusted covariates in each of the included studies are listed in Table 3. A total of 12 studies evaluated the impact of maternal age and most studies evaluated maternal smoking during pregnancy, offspring sex, maternal educational level, parity and year of birth. Adjustment for birthweight, gestational age, weight gain during pregnancy, maternal country, paternal BMI, children BMI and parental ADHD occurred less often.

**Table 2.** Quality assessment by the Newcastle-Ottawa Scale

Study	Study design	Selection	Comparability	Outcome	Total
1) Rodriguez A (2008)	Cohort	****	*	***	8
2) Rodriguez A (2010)	Cohort	****	*	***	8
3) Brion MJ (2011)	Cohort	***	*	***	7
4) Buss C (2012)	Cohort	***	-	***	6
5) Hinkle SN (2013)	Cohort	***	**	**	7
6) Chen Q (2014)	Cohort	****	**	***	9
7) Van Mil (2014)	Cohort	***	**	**	7
8) Jo H (2015)	Cohort	***	*	**	6
9) Andersen CH (2017)	Cohort	****	*	***	8
10) Musser ED (2017)	Cohort	****	**	***	9
11) Casas M (2017)	Cohort	***	**	***	8
12) Daraki V (2017)	Cohort	***	*	***	7
13) Mina TH (2017)	Cohort	****	*	***	8
14) Mikkelsen SH (2017)	Cohort	****	**	***	9

**Table 3.** Confounders and risk factors evaluated in studies of maternal overweight or obesity and risk of ADHD in offspring

Group	Variables	1^a^	2	3	4	5	6	7	8	9	10	11	12	13	14
Parental characteristics	Maternal age	×	×			×	×	×	×	×	×	×	×	×	×
	Paternal age									×					×
	Race				×	×			×						
	Birth country	×					×						×		
	Social class/status (base on education and occupation)			×						×		×			×
	Family income/poverty			×		×			×					×	
	Family structure during pregnancy		×												
	Family structure at follow-up	×	×												
	Maternal employment status during pregnancy/follow-up											×			
	Marital status/cohabitation						×		×						×
	Maternal smoking(during pregnancy)	×	×	×		×	×	×	×	×			×	×	×
	Weight gain during pregnancy	×	×		×				×		×				
	Gestational diabetes							×	×					×	
	Maternal IQ				×							×			
	Life events (e.g. interpersonal loss, personal financial problems relocation and serious illness within the previous year)		×												
	Maternal anxiety													×	
	Depressive symptoms at follow-up				×									×	
	Depressive symptoms during/after pregnancy		×						×						
	Paternal education			×								×			
	Maternal education	×	×	×	×	×	×	×	×			×	×		
	Paternal BMI			×								×	×		×
Pregnancy-related	Parity/birth order				×	×	×	×	×		×	×	×	×	×
	Apgar score 1 minute after birth							×							
	Mode of delivery							×							
	Pre-eclampsia							×							
	Folic acid supplementation							×							
	Breastfeeding duration								×			×			
	Daycare attendance											×			
	Obstetric risk				×										
Offspring characteristics	Birthweight	×	×		×				×	×				×	
	Gestational age	×	×		×					×	×			×	
	Infant sex	×	×		×	×	×	×	×	×	×	×	×	×	×
	Child BMI percentile/overweight		×		×	×			×						
	Child physical activity/TV hours					×						×			
	Child’s enrichment (read or special lessons)					×			×						
	Year of kindergarten entry					×									
ADHD-related	Age at assessment/ year of birth				×	×	×		×		×	×	×	×	×
	Paternal or/and maternal hyperactivity/ADHD		×								×				×
	Maternal psychiatric diagnoses							×		×					

a Number of studies same as Table 2.

#### Meta-analysis

**Figure 3:**
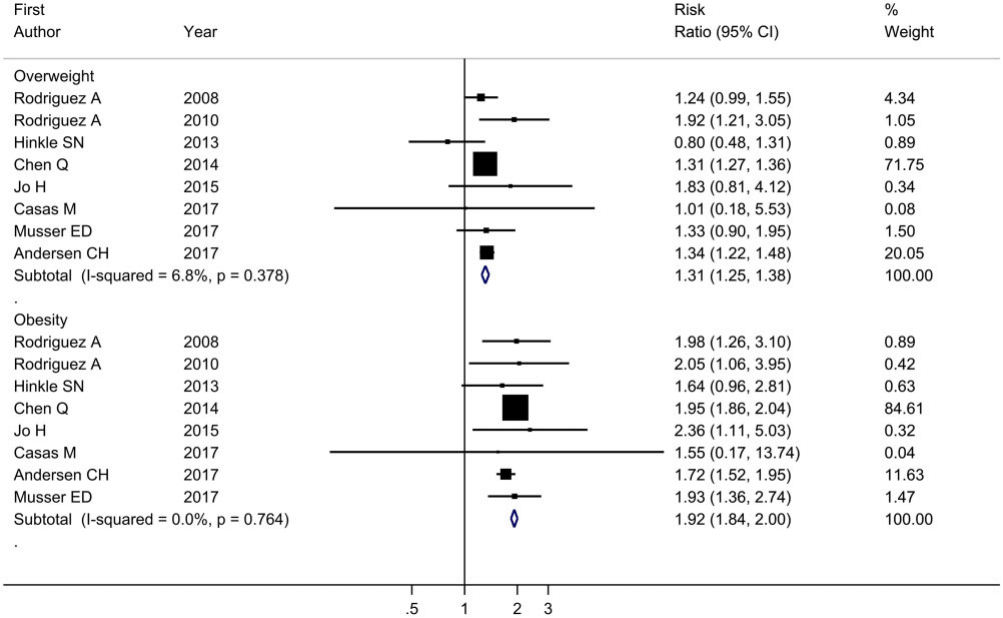
Forest plot of all studies describing maternal pre-pregnancy overweight (BMI 25–29.99) or obesity (BMI* *≥* *30.0) and crude risk of ADHD in offspring. ADHD, attention-deficit/hyperactivity disorder; BMI, body mass index.

**Figure 4:**
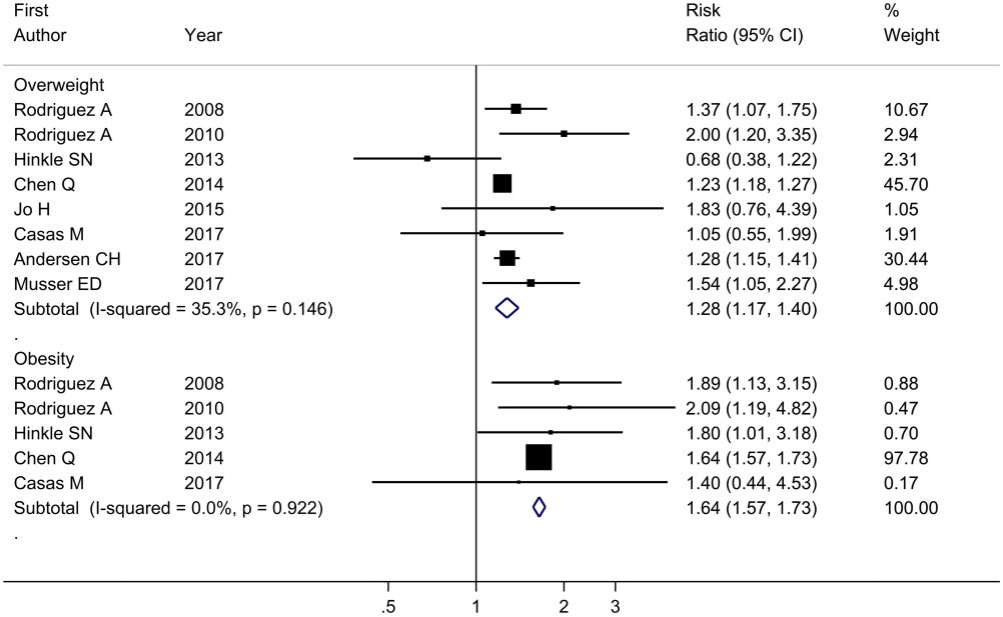
Forest plot of all studies describing maternal pre-pregnancy overweight (BMI 25–29.99) or obesity (BMI* *≥* *30.0) and adjusted risk of ADHD in offspring. ADHD, attention-deficit/hyperactivity disorder; BMI: body mass index.

**Figure 5:**
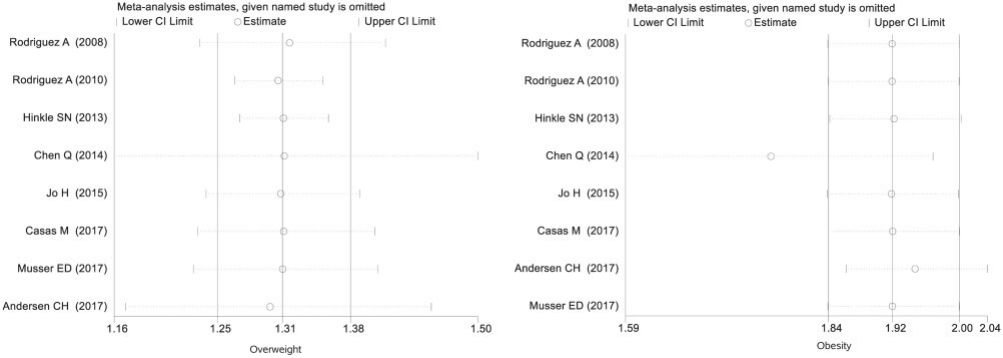
Results of leave-one-out sensitivity analysis. The vertical axis shows the omitted study. Every circle indicates the pooled RR when the left study is omitted in this meta-analysis. The two ends of every broken line represent the respective 95% confidence interval.

The meta-analysis showed increased risk of ADHD in offspring born to mothers with overweight (RR = 1.31, 95% CI = 1.25–1.38, *I*^2^ = 6.80%) and obesity (RR = 1.92, 95% CI = 1.84–2.00, *I*^2^ = 0.0%) compared with those born to normal-weight mothers (Figure 3). The adjusted RRs were somewhat attenuated for both maternal overweight (RR = 1.28, 95% CI = 1.17–1.40, *I*^2^ = 35.3%) and obesity (RR = 1.64, 95% CI = 1.47–1.73, *I*^2^ = 0.0%), but the same pattern was observed (Figure 4). Among the studies estimating the association between maternal pre-pregnancy overweight and ADHD in offspring, the pooled RRs of the leave-one-study-out analysis were similar to those in the main analysis. When we repeated the analysis among studies evaluating the association between maternal pre-pregnancy obesity and ADHD in offspring, the overall estimate of the RR was slightly decreased to 1.77 (95% CI = 1.59–1.97, *I*^2^ = 0.0%) after excluding the previous study based on a large Swedish sample. However, the direction of the association was still stable and the effect size was close to the pooled RR in the main analysis (Figure 5). Using definitions of ADHD other than teacher-rated inattention symptoms produced similar results to those in the main analysis (Table 4).

**Table 4.** Sensitivity analyses among studies with different ADHD definitions

Outcome	Overweight		Obesity	
	RR (95% CI)	*I* ^2^ (*P*-value)	RR (95% CI)	*I* ^2^ (*P*-value)
Teacher-rated AD	1.31 (1.25–1.38)	6.8% (0.38)	1.92 (1.84–2.00)	0.0% (0.76)
Teacher-rated HD	1.30 (1.25–1.36)	4.3% (0.40)	1.81 (1.62–2.03)	36.7% (0.13)
Mother-rated AD	1.31 (1.27–1.35)	0.0% (0.58)	1.85 (1.69–2.02)	21.4% (0.26)
Mother-rated HD	1.25 (1.14–1.36)	14.9% (0.31)	1.67 (1.40–2.00)	87.0% (0.00)

ADHD, attention-deficit/hyperactivity disorder; AD, attention-deficit symptoms; HD, hyperactivity symptom; RR, risk ratios; CI, confidence interval.

**Figure 6:**
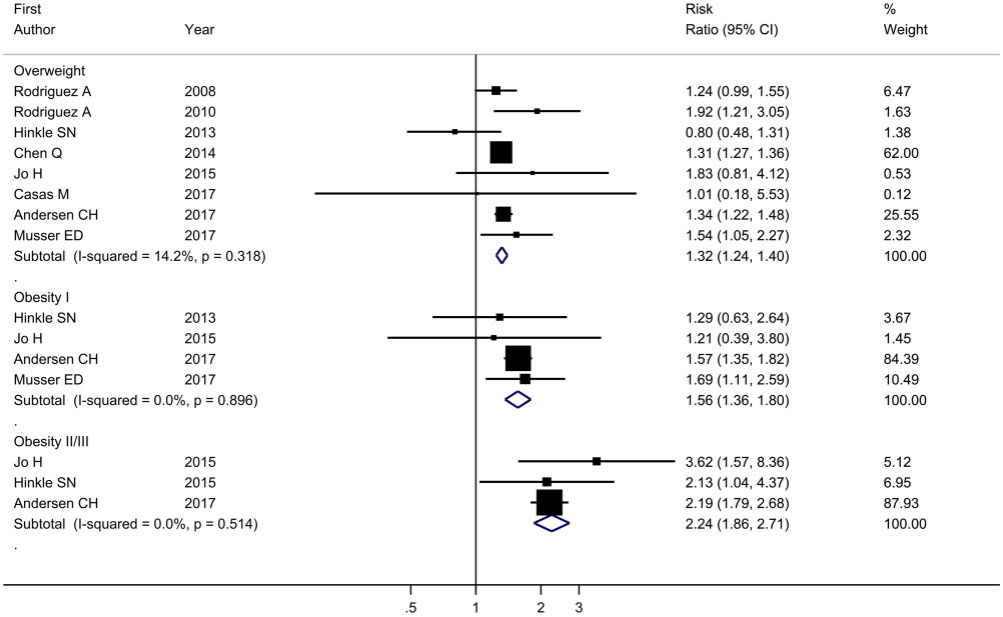
Forest plot of studies describing maternal pre-pregnancy overweight (BMI 25–29.99), obesity I (BMI 30–34.99) and obesity II/III (BMI* *≥* *35.0) and crude risk of ADHD in offspring. ADHD, attention-deficit/hyperactivity disorder; BMI, body mass index.

Subgroup analyses based on different measurements of ADHD (CBCL/SDQ/self-reported), different informants (parents/teachers) and time of maternal BMI (pre-pregnancy/early pregnancy) suggested that the association between maternal overweight or obesity and risk of ADHD in offspring was robust. However, the stratified analyses of different informants of children’s ADHD symptoms generated imprecise estimates, as only two previous studies^43^^,^^44^ provided information on parent-rated ADHD of offspring (Table 5). When we further repeated the main analysis among the studies that reported results for different obesity groups (obesity class I, obesity class II/III), the risk of having offspring with ADHD was still elevated for obesity I (RR = 1.56, 95% CI = 1.36–1.80, *I*^2^ = 0.0%) and obesity II/III (RR = 2.24, 95% CI = 1.86–2.71, *I*^2^ = 0.0%) mothers (Figure 6).

**Table 5.** Summary of results from sensitivity analyses and subgroup analyses

			Overweight		Obesity	
Group	No. of studies	Sample size	RR (95% CI)	*I* ^2^ (*P*-value)	RR (95% CI)	*I* ^2^ (*P*-value)
Crude effect size	8	784 804	1.31 (1.25–1.38)	6.8% (0.38)	1.92 (1.84–2.00)	0.0% (0.76)
Adjusted effect size	8	784 804	1.28 (1.17–1.40)	35.3% (0.15)	–	–
	5	696 919	–	–	1.64 (1.47–1.73)	0.0% (0.92)
ADHD diagnosis	5	766 717	1.31 (1.24–1.38)	12.5% (0.33)	1.93 (1.83–2.01)	1.2% (0.40)
ADHD symptoms	3	18 087	1.42 (1.03–1.95)	30.8% (0.24)	1.99 (1.38–2.87)	0.0% (0.97)
Teacher-rated ADHD diagnosis/symptom	3	18 087	1.42 (1.03–1.95)	30.8% (0.24)	1.99 (1.38–2.87)	0.0% (0.97)
Parent-rated ADHD diagnosis/symptom	2	6511	1.31 (0.51–2.53)	33.3% (0.22)	1.85 (1.20–2.87)	0.0% (0.44)
Records from registers	3	761 517	1.31 (1.27–1.36)	0.00% (0.91)	1.87 (1.71–2.05)	41.7% (0.18)
Measured BMI	3	20 942	1.38 (1.10–1.73)	28.2% (0.25)	1.96 (1.52–2.53)	0.0% (0.98)
Self-reported BMI	5	763 862	1.31 (1.24–1.39)	14.0% (0.32)	1.91 (1.82–2.01)	2.0% (0.40)
Pre-pregnancy BMI	5	94 912	1.29 (1.10–1.51)	30.0% (0.24)	1.75 (1.56–1.96)	0.0% (0.90)
Early-pregnancy BMI	3	689 892	1.33 (1.18–1.40)	13.6% (0.33)	1.95 (1.86–2.04)	0.0% (0.98)
Without Sweden population	7	111 172	1.32 (1.16–1.50)	19.4% (0.28)	1.77 (1.59–1.97)	0.0% (0.96)

ADHD, attention-deficit/hyperactivity disorder; BMI, body mass index; RR, risk ratio; CI: confidence interval.

**Table 6.** Publication bias among the included studies

	Egger’s test	Begg’s test	Funnel plot
Overall	*P* = 0.621	*P* = 0.787	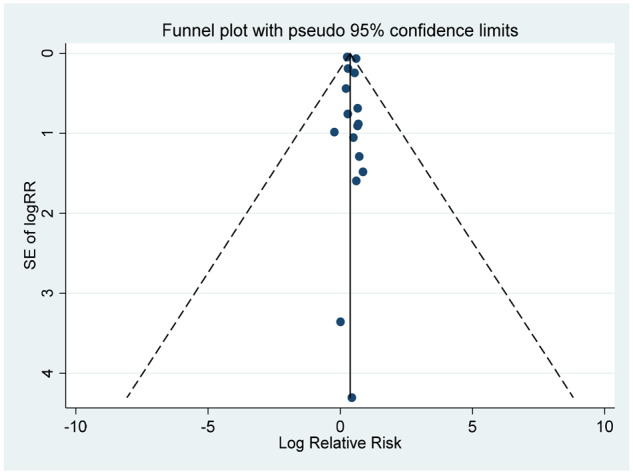
Overweight	*P* = 0.879	*P* = 0.621	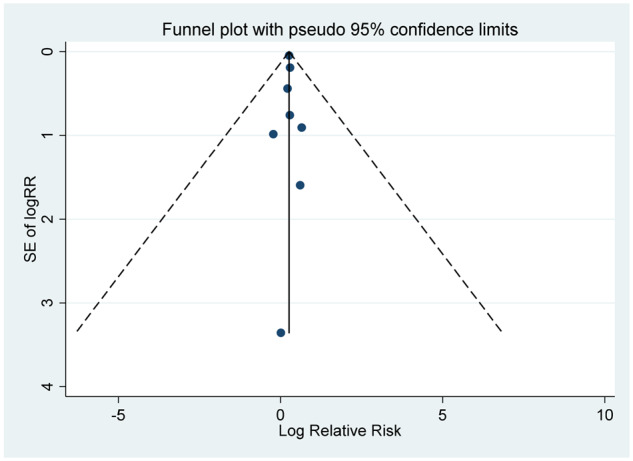
Obesity	*P* = 0.685	*P* = 0.805	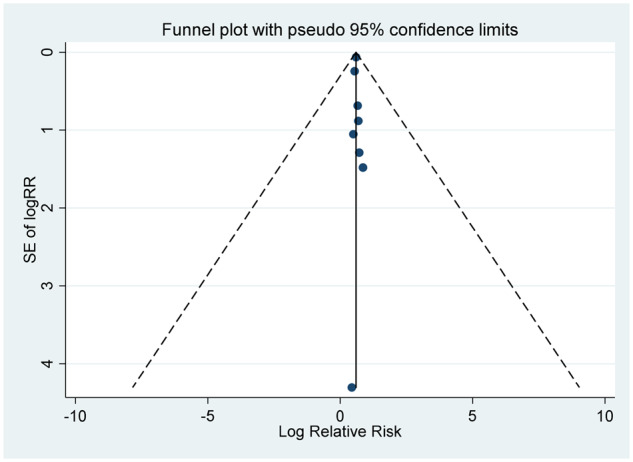

#### Publication bias

There was no evidence of publication bias according to Begg’s test and Egger’s test (all *P *>* *0.5) and Funnel plots (Table 6).

### Nationwide population-based cohort study

In total, 43 916 (4.52%) offspring with a diagnosis of ADHD were identified in the entire cohort. Table 7 shows the distribution of offspring and maternal covariates. Offspring exposed to maternal overweight or obesity were more likely to be of late parity (*P *<* *0.01) and to have mothers who were born outside Sweden (*P *<* *0.01), smoked during pregnancy (*P *<* *0.01), had lower education (*P *<* *0.01) and did not live together with the biological father at childbirth (*P *<* *0.01) (Supplementary Table 2, available as Supplementary data at *IJE* online).

**Table 7.** Demographic characteristics of offspring and their mothers

Covariates	Entire cohort (*N* = 971 501)*N* (%)	First cousins (*N* = 155 841)*N* (%)	Full siblings (*N *= 463 474)*N* (%)
Offspring sex			
Male	496 904 (51.15)	79 515 (51.02)	237 944 (51.34)
Female	474 597 (48.85)	76 326 (48.98)	225 530 (48.66)
Birth order			
1	408 924 (42.09)	96 165 (61.71)	171 508 (37.00)
2	358 659 (36.92)	37 239 (23.90)	195 983 (42.29)
3	142 298 (14.65)	16 402 (10.52)	66 305 (14.31)
4+	61 620 (6.34)	6035 (3.87)	29 678 (6.40)
Offspring year of birth			
1992–95	329 692 (33.94)	70 746 (45.40)	131 283 (28.33)
1996–99	273 005 (28.10)	40 981 (26.30)	159 420 (34.40)
2000–04	368 804 (37.96)	44 114 (28.31)	172 771 (37.28)
Mother’s country of birth			
Sweden	819 738 (84.38)	148 292 (95.16)	396 810 (85.62)
Denmark, Finland, Iceland or Norway	23 436 (2.41)	2355 (1.51)	9519 (2.05)
Other	128 327 (13.21)	5194 (3.33)	57 145 (12.33)
Maternal education			
≤9 years	81 510 (8.57)	10 706 (6.96)	33 088 (7.26)
10–12 years	455 747 (47.91)	76 153 (49.48)	214 445 (47.07)
Postgraduate education	414 027 (43.52)	67 049 (43.56)	208 055 (45.67)
Maternal age at delivery			
≤19	18 934 (1.95)	3803 (2.44)	5911 (1.28)
20–24	157 079 (16.17)	31 082 (19.94)	76 319 (16.47)
25–29	347 824 (35.80)	61 774 (39.64)	178 697 (38.56)
30–34	302 558 (31.14)	43 207 (27.73)	146 282 (31.56)
≥35	145 106 (14.94)	15 975 (10.25)	56 265 (12.14)
Smoking during pregnancy			
No	813 931 (85.44)	128 377 (84.03)	413 009 (89.11)
1–9 cigarettes per day	91 977 (9.66)	16 289 (10.66)	34 155 (7.37)
≥10 cigarettes per day	46 707 (4.90)	8110 (5.31)	16 310 (3.52)
Cohabitation with child’s father at childbirth			
Yes	893 754 (95.05)	142 142 (94.80)	452 048 (97.53)
No	46 523 (4.95)	7793 (5.20)	11 426 (2.47)

Missing values: in the entire cohort, 20 217 individuals missed data for maternal highest education, 18 886 for smoking during pregnancy, 31 224 for cohabitation status; in sibling samples, 7886 individuals missed data for maternal highest education. In cousin samples, 1993 individuals missed data for maternal highest education, 3065 for smoking during pregnancy, 5906 for cohabitation status.

#### Main analysis

At the population level, the overall crude risk of ADHD in offspring was elevated in mothers with overweight or obesity (Table 8). The more severe the obesity, the higher the hazard of ADHD, with a *P*-value for trend <0.01. The HRs for overweight and obese mothers were 1.30 (95% CI = 1.28–1.34) and 1.92 (95% CI = 1.87–1.98), respectively. Mothers with obesity class I, II and III had HRs of 1.82 (95% CI = 1.76–1.88), 2.24 (95% CI = 2.12–1.38) and 2.87 (95% CI = 2.50–3.31), respectively. After adjustment for measured covariates, the associations of maternal pre-pregnancy overweight (HR_overweight_ = 1.21, 95% CI = 1.19–1.25) and obesity (HR_obesity_ = 1.60, 95% CI = 1.55–1.65) with ADHD in offspring were slightly attenuated. Mothers with obesity class I, II and III had adjusted HRs of 1.53 (95% CI = 1.48–1.59), 1.78 (95% CI = 1.67–1.89) and 2.20 (95% CI = 1.89–2.57), respectively. Consistently with the analyses at the population level, crude HRs attenuated when adjusting for measured covariates in the first-born full-cousin comparisons and full-sibling comparisons (Supplementary Table 3, available as Supplementary data at *IJE* online).

**Table 8.** Hazard ratios for ADHD among offspring exposed to different levels of maternal pre-pregnancy BMI

Exposure	Entire populationHR(95% CI)				First-born full cousinsHR (95% CI)		Full siblingsHR (95% CI)	
	Unadjusted	*P*	Adjusted^a^	*P*	Adjusted^b^	*P*	Adjusted^c^	*P*
Pre-pregnancy normal weight	Reference		Reference		Reference			
Pre-pregnancy overweight	1.30 (1.28–1.34)	0.00	1.21 (1.19–1.25)	0.00	1.10 (0.98–1.23)	0.13	1.01 (0.92–1.11)	0.80
Pre-pregnancy obesity	1.92 (1.87–1.98)	0.00	1.60 (1.55–1.65)	0.00	1.44 (1.22–1.70)	0.00	1.10 (0.94–1.27)	0.24
** **Obesity Class I	1.82 (1.76–1.88)	0.00	1.53 (1.48–1.59)	0.00	1.38 (1.15–1.65)	0.00	1.10 (0.94–1.29)	0.24
** **Obesity Class II	2.24 (2.12–1.38)	0.00	1.78 (1.67–1.89)	0.00	1.49 (1.08–2.05)	0.01	1.06 (0.82–1.36)	0.66
** **Obesity Class III	2.87 (2.50–3.31)	0.00	2.20 (1.89–2.57)	0.00	1.41 (0.53–3.75)	0.49	1.70 (0.99–2.91)	0.05
*P*-value for trend^d^	<0.0001		<0.0001		<0.0001		0.267	
Continuous BMI	1.04 (1.04–1.05)	0.00	1.04 (1.03–1.04)	0.00	1.03 (1.02–1.04)	0.00	1.00 (0.99–1.03)	0.35

a *N = *903 824. Adjusted for offspring sex, birth order, year of birth, mother’s country of birth, highest maternal education, maternal age at delivery, smoking during pregnancy and cohabitation with child’s father at childbirth.

b *N = *146 796. Adjusted for offspring sex, birth order, year of birth, mother’s country of birth, highest maternal education, maternal age at delivery, smoking during pregnancy and cohabitation with child’s father at childbirth, and shared familial confounding within first-born cousins.

c *N = *463 474. Adjusted for offspring sex, birth order, year of birth, maternal age at delivery, smoking during pregnancy and cohabitation with child’s father at childbirth, and shared familial confounding within full siblings.

d *P*-value for trend was tested among groups: normal weight, overweight, obesity I, obesity II, obesity III.

ADHD, attention-deficit/hyperactivity disorder; BMI, body mass index; HR, hazard ratio; CI, confidence interval.

The associations were further attenuated in first-born maternal full-cousin-comparison models when taking measured covariates and unmeasured factors shared by first cousins into consideration (HR_overweight_ = 1.10, 95% CI = 0.98–1.23; HR_obesity_ = 1.44, 95% CI = 1.22–1.70). Sibling-comparison models showed that the observed association at the entire population level were largely attenuated towards the null (HR_overweight_ = 1.01, 95% CI = 0.92–1.11; HR_obesity_ = 1.10, 95% CI = 0.94–1.27). The associations of maternal pre-pregnancy obesity class I–III with ADHD in offspring were also largely attenuated and the dose–response association no longer existed in the sibling-comparison analysis, but the point estimate and the upper confidence interval for obesity class III indicated a potential association with ADHD in offspring (HR = 1.70, 95% CI = 0.99–2.91).

When analysing BMI as a continuous trait, the attenuated effect within full cousins (HR_BMI_ = 1.03, 95% CI = 1.02–1.04) and the null effect within full siblings (HR_BMI_ = 1.00, 95% CI = 0.99–1.03) were replicated, demonstrating the robustness of our main results (Table 8).

#### Sensitivity analyses

First, analyses restricted to first- and second-born sibling pairs yielded similar results to those in the main analyses (HR_overweight_ = 1.00, 95% CI = 0.91–1.11; HR_obesity_ = 1.04, 95% CI = 0.88–1.24; HR_obesity I_ = 1.05, 95% CI = 0.88–1.24; HR_obesity II_ = 0.97, 95% CI = 0.73–1.28; and HR_obesity III_ = 1.73 , 95% CI = 0.94–3.16), indicating that the results of the main analysis are robust (Table 9). To further explore the effect modification by birth order, we conducted stratified analyses based on first- and second-born siblings. Comparing to first-born siblings (HR_overweight_ = 1.29, 95% CI = 1.22–1.37; HR_obesity_ = 1.66, 95% CI = 1.52–1.81), similar associations were found in second-born siblings (HR_overweight_ = 1.27, 95% CI = 1.20–1.34; HR_obesity_ = 1.78, 95% CI = 1.66–1.91) (Supplementary Table 4, available as Supplementary data at *IJE* online), indicating the modification by birth order was of limited importance. Second, weight gain and weight loss between two pregnancies may indicate different biological mechanisms and the effect of familial confounding may differ across different types of between-pregnancy variation in BMI.^19^ However, similar associations were observed in the population level and stratified sibling comparisons when we conducted the bidirectional case-crossover analysis (Table 10), suggesting that the influence of changing weight status and carryover effects between two pregnancies was of limited importance. Third, to rule out potential confounding by bariatric surgery, we restricted the analysis to those who had never had bariatric surgeries before delivery (*N* = 957 473). All results were consistent with the main analyses among mothers with overweight or obesity at the population level, first-born maternal full-cousin comparisons and first- and second-born sibling pairs (Supplementary Table 5, available as Supplementary data at *IJE* online).

**Table 9.** Hazard ratios for ADHD based on first-born and second-born siblings exposed to different levels of maternal pre-pregnancy BMI

	Adjusted HR (95% CI)^a^	*P*-value
Pre-pregnancy normal weight	Reference	
Pre-pregnancy overweight	1.00 (0.91–1.11)	0.98
Pre-pregnancy obesity	1.04 (0.88–1.24)	0.63
Obesity class I	1.05 (0.88–1.24)	0.61
Obesity class II	0.97 (0.73–1.28)	0.82
Obesity class III	1.73 (0.94–3.16)	0.08
*P*-value for trend ^b^	0.39	

a *N = *432* *168. Adjusted for offspring sex, birth order, year of birth, maternal age at delivery, smoking during pregnancy and cohabitation with child’s father at childbirth, and shared familial confounding within full siblings.

b *P*-value for trend was tested among groups: normal weight, overweight, obesity I, obesity II, obesity III.

ADHD, attention-deficit/hyperactivity disorder; BMI, body mass index; HR, hazard ratio; CI, confidence interval.

**Table 10.** Hazard ratios for ADHD based on mothers with different patterns of variation in BMI

BMI category		No. of pairs	Difference in BMI	Entire population^a^		Full siblings^b^	
First pregnancy	Second pregnancy		Mean (SD)	HR (95% CI)	*P*-value	HR (95% CI)	*P*-value
Normal	Normal	131 765	0.44 (1.24)	1.02 (1.01–1.03)	0.00	1.02 (0.98–1.06)	0.30
Normal	Overweight/obese	25 317	2.96 (1.85)	1.03 (1.01–1.06)	0.01	1.03 (0.97–1.09)	0.29
Overweight/obese	Normal	6589	2.47 (1.99)	1.03 (0.98–1.08)	0.31	0.98 (0.88–1.10)	0.77
Overweight/obese	Overweight/obese	52 763	1.20 (2.25)	1.05 (1.04–1.06)	0.00	1.00 (0.97–1.03)	0.92

a Adjusted for offspring sex, birth order, year of birth, maternal age at delivery, smoking during pregnancy and cohabitation with child’s father at childbirth.

b Adjusted for offspring sex, birth order, year of birth, maternal age at delivery, smoking during pregnancy and cohabitation with child’s father at childbirth, and shared familial confounding within full siblings.

ADHD, attention-deficit/hyperactivity disorder; SD, standard deviation; BMI, body mass index; HR, hazard ratio; CI, confidence interval.

## Discussion

By combining a systematic review, meta-analysis based on previous studies with a nationwide population-based cohort study with sibling and cousin comparisons, we rigorously explored the association between maternal pre-pregnancy overweight/obesity and risk of ADHD in offspring, assessing dose–response effects and the role of unmeasured confounding. The meta-analysis revealed a positive association between maternal pre-pregnancy BMI and risk of ADHD in offspring. Similar results were observed in the nationwide population-based cohort study based on Swedish registers after adjusting for measured covariates. However, in cousin and sibling comparisons, the associations were largely attenuated towards the null, suggesting that the association between maternal pre-pregnancy BMI and risk of ADHD in offspring could be largely ascribed to unmeasured familial confounding.

Consistently with the results from previous meta-analysis studies,^7^^,^^50^ we also found a positive association between maternal pre-pregnancy overweight/obesity and ADHD in offspring. However, our study strengthens and extends previous findings in three ways. First, we found robust results across different definitions and assessment approaches of both overweight/obesity and ADHD. Second, previous meta-analytic findings need to be interpreted with caution, since these meta-analysis studies suffered from important methodological limitations: (i) the estimates may not be corrected for including more than one estimate from the same study when pooled estimates were calculated,^7^^,^^50^ which may introduce over-representation bias; (ii) some studies in these meta-analyses were based on highly selected samples, such as a high-risk population with ADHD prevalence of 11.0%,^51^ alcohol and marijuana cohort (only includes women who drank more than three drinks per week or smoked more than two joints per month),^52^ a cohort in which joint effects of diabetes and severely obesity were explored^53^ or only preterm birth samples,^54^ which may limit generalizability; (iii) several recent and important studies were not included in the Sanchez’s and Jenabi’s meta-analysis work.^43^^,^^46^ Third, combined with a nationwide family-based cohort study, we further evaluated the results from the pooled estimates of previous studies by adjusting for measured confounding identified via a DAG and unmeasured confounding by using various genetically informative designs—an approach similar to that used in Cortese *et al*.^17^ Therefore, we could further explore potential alternative explanations for the observed associations.

Similarly to previous sibling-comparison studies,^19^^,^^20^ we found that the association between maternal pre-pregnancy overweight/obesity and increased risk of ADHD in offspring was largely explained by unmeasured familial confounders. Maternal pre-pregnancy overweight/obesity probably represents, at least in part, a genetic predisposition to ADHD in offspring, as both population-based familial co-aggregation studies^12^ and a recent genome-wide association study^13^ have suggested a genetic overlap between overweight/obesity and ADHD. Importantly, even though twin studies consistently have demonstrated that shared environmental factors probably are of limited importance in ADHD,^3^ influences from such factors cannot be ruled out completely.^55^ We were able to extend the previous family-based quasi-experimental studies (i.e. sibling-comparison studies)^19^^,^^20^ in three important ways. First, our bidirectional case-cross analysis indicated that carryover effects between two pregnancies were of limited importance. Second, findings from both sibling comparisons and first-cousin comparisons consistently suggested the presence of unmeasured familial confounding indicating that findings from sibling comparisons generalize to other settings. This is important given that women who varied in their weight status between pregnancies might not be comparable to women who were constantly overweight/obese. Third, with the largest sample size, we could further explore and confirm the dose–response associations of maternal pre-pregnancy obesity class I–III with ADHD in offspring.

### Limitations

The results of the meta-analysis should be interpreted with caution. First, the assessment of ADHD varied across the studies. However, the subgroup analyses on the different ADHD measurements suggested that the results were robust independently of the assessment approaches of the studies included in the meta-analysis. Second, three studies based on Nordic national medical registers used maternal early-pregnancy BMI as a proxy for pre-pregnancy BMI. Although early gestational weight and pre-pregnancy weight were highly correlated in a previous study,^49^ somewhat lower overall RRs were found among studies with pre-pregnancy overweight/obesity as the exposure compared with those that used early-pregnancy overweight/obesity as the exposure. Thus, the associations reported in the current meta-analysis might be overestimated. Third, we were not able to calculate a pooled RR among studies using sibling comparisons, as the two available studies^19^^,^^20^ used different methods [Cox proportional-hazards model and generalized estimating equations (GEE)], which cannot be combined. Therefore, we further compared the pooled RRs obtained from current meta-analysis with those observed from an original cohort study with a family-based quasi-experimental study design. Fourth, all included studies were conducted in Europe (in particular, the large cohorts in Nordic countries) and the US, which limit generalizability to other populations across the world. We therefore suggest future studies to examine the associations using different samples, especially in countries outside Europe and the US. Future studies with different study designs, e.g. intergenerational Mendelian randomization or children-of-twins design, are also needed to triangulate our findings.

The nationwide population-based cohort study also had limitations. First, as already discussed and similarly to previous register-based observational studies included in our meta-analysis,^19^^,^^34^^,^^35^ we used early-pregnancy BMI (around 10 weeks of gestation) as a proxy for pre-pregnancy BMI. Based on evidence from the above meta-analysis, we might overestimate the magnitude of the association, although this overestimate was unlikely to affect our conclusion on sibling comparisons. Second, the current cohort study suffered from common limitations among register-based studies, such as measurement errors using information from the medical records and limited availability of measured confounding variables. Third, BMI may not be an accurate proxy for total body fat and overweight/obesity-related metabolic conditions.^56^ Future studies would benefit from using more direct measurement/observation/diagnosis of obesity or specific maternal pre-pregnancy conditions (e.g. metabolic syndrome). Fourth, sibling and cousin comparisons are not able to control for time-varying family-wide confounders, like maternal age, which may, although not necessarily, invalidate unmeasured familial confounding as the main explanation for the observed association. Another limitation of the sibling-comparison design is the loss of power to make definitive conclusions about the highest level of obesity (obesity class III), as there were only nine families with siblings discordant for maternal pre-pregnancy extreme obesity (obesity class III), of which only one family was also discordant for ADHD (Supplementary Table 6, available as Supplementary data at *IJE* online). That is, this double-discordant family contributed with the main information to the analysis of obesity class III and, in the adjusted Cox proportional-hazards model, siblings discordant for exposure time (e.g. differences in the length of follow-up) or other covariates are also informative. Fifth, despite the large sample size, we cannot completely rule out a potential causal link from maternal pre-pregnancy overweight/obesity to ADHD in offspring. Compared with the previous Swedish sibling study^19^ (HR_obesity_ = 1.15, 95% CI = 0.85–1.56), we found a lower magnitude of the HR and a narrower confidence interval among obese women in sibling comparisons (HR_obesity_ = 1.10, 95% CI = 0.94–1.27), but the upper limit of the 95% CI was still non-negligible, especially in moderate (obesity class II) (HR _upper 95%CI_ = 1.36) and extremely obese (obesity class III) (HR _upper 95%CI_ = 2.91) women. Nonetheless, any causal relationship is unlikely to be as strong as that found in the meta-analysis. Future work is needed to explore the nature of the familial confounding and the potential risks associated with severe pre-pregnancy obesity (e.g. obesity class III).

In conclusion, there is an association between maternal pre-pregnancy overweight/obesity and ADHD in offspring, but this association is largely ascribable to unmeasured familial confounding and not a strong causal relationship. Our findings highlight the importance of accounting for unmeasured familial confounders in risk-factor studies of ADHD in offspring. Future studies need to elucidate the genetic and environmental origins of the unmeasured confounding and more studies with different methods and designs, in various populations or focusing on sever maternal obesity, are still needed to replicate and build upon our findings.

## Supplementary Data

Supplementary data are available at *IJE* online.

## Funding

This work was supported financially by the Swedish Research Council (Grant No. 2018–02599), the ***S***wedish ***I***nitiative for Research on ***M***icrodata in the ***S***ocial ***A***nd ***M***edical Sciences (SIMSAM) framework (Grant No. 340–2013-5867), and European Union’s Horizon 2020 research and innovation programme (Grant Aggreement No. 667302).

## Conflict of Interest

Dr Larsson has served as a speaker for Evolan and Shire and has received research grants from Shire; Dr Cortese reports receiving reimbursement for travel and accommodation expenses from the Association for Child and Adolescent Central Health (ACAMH), a non-profit organization, Healthcare, British Association of Psychopharmacology (BAP) and the Canadian Alliance ADHD Resource (CADDRA) in relation to lectures that he delivered on ADHD, all outside the submitted work.

## Supplementary Material

dyaa040_Supplementary_Data
