# Supplementary material for: Maternal pre-pregnancy overweight/obesity and the risk of attention-deficit/hyperactivity disorder in offspring: a systematic review, meta-analysis and quasi-experimental family-based study
Source: Int J Epidemiol. 2020 Apr 26;49(3):857–75. doi: 10.1093/ije/dyaa040 (PMC7394963; doi:10.1093/ije/dyaa040)
Supplement: dyaa040_Supplementary_Data [file dyaa040_supplementary_data.docx]

***Supplemental Tables***

**Table S1** Search strategy and results from each electronic database (updated to 2018-12-31)

| **Source** |  | **Search Strategy** | **Results** |
| --- | --- | --- | --- |
| **Pubmed** |  |  |  |
|  | #1 | Mothers[MeSH] OR Pregnancy[MeSH] | [718 111](https://www.ncbi.nlm.nih.gov/pubmed/?cmd=HistorySearch&querykey=2) |
|  | #2 | Prenatal[Title/Abstract] OR Perinatal[Title/Abstract] OR Maternal[Title/Abstract] OR Gestational[Title/Abstract] OR Pre-pregnancy[Title/Abstract] OR Pregnan*[Title/Abstract] | 535 796 |
|  | #3 | “Body Weights and Measures”[MeSH] OR Obesity[MeSH] OR “Body Weight”[MeSH] | [418 256](https://www.ncbi.nlm.nih.gov/pubmed/?cmd=HistorySearch&querykey=4) |
|  | #4 | Weight[Title/Abstract] OR “body mass index”[Title/Abstract] OR BMI[Title/Abstract] OR Obes*[Title/Abstract] OR Adipos*[Title/Abstract] OR overweight[Title/Abstract] OR “body weight”[Title/Abstract] | [641 107](https://www.ncbi.nlm.nih.gov/pubmed/?cmd=HistorySearch&querykey=16) |
|  | #5 | Child[MeSH] | [1 797 221](https://www.ncbi.nlm.nih.gov/pubmed/?cmd=HistorySearch&querykey=12) |
|  | #6 | child*[Title/Abstract] OR offspring[Title/Abstract] | [1 169 301](https://www.ncbi.nlm.nih.gov/pubmed/?cmd=HistorySearch&querykey=10) |
|  | #7 | “Attention deficit disorder with hyperactivity”[MeSH] | [25 660](https://www.ncbi.nlm.nih.gov/pubmed/?cmd=HistorySearch&querykey=20) |
|  | #8 | ADHD [Title/Abstract] OR ADDH [Title/Abstract] OR ADD [Title/Abstract] OR Attention [Title/Abstract] OR Inattention [Title/Abstract] OR Hyperactiv*[Title/Abstract] OR Impulsiv* [Title/Abstract] OR Hyperkinetic disorder [Title/Abstract] | 314 467 |
|  | #9 | Case-control studies[MeSH] OR Cohort Studies[MeSH] OR  Epidemiologic Studies[MeSH] OR Epidemiology [Mesh:NoExp] | [2 228 405](https://www.ncbi.nlm.nih.gov/pubmed/?cmd=HistorySearch&querykey=26) |
|  | #10 | Case-Control Stud* [Title/Abstract] OR Cohort Stud* [Title/Abstract] OR Epidemiologic Stud* [Title/Abstract] OR  Case-control [Title/Abstract] OR Cohort[Title/Abstract] OR Epidemiolog*[Title/Abstract] OR Observational[Title/Abstract] OR Population [Title/Abstract] OR Longitudinal [Title/Abstract] OR Prospective[Title/Abstract] OR Follow-up [Title/Abstract] OR Retrospective[Title/Abstract] | [2 594 863](https://www.ncbi.nlm.nih.gov/pubmed/?cmd=HistorySearch&querykey=24) |
|  | #11 | #1 OR #2 | [845 207](https://www.ncbi.nlm.nih.gov/pubmed/?cmd=HistorySearch&querykey=8) |
|  | #12 | #3 OR #4 | [786 838](https://www.ncbi.nlm.nih.gov/pubmed/?cmd=HistorySearch&querykey=14) |
|  | #13 | #5 OR #6 | [2 092 061](https://www.ncbi.nlm.nih.gov/pubmed/?cmd=HistorySearch&querykey=22) |
|  | #14 | #7 OR #8 | [318 412](https://www.ncbi.nlm.nih.gov/pubmed/?cmd=HistorySearch&querykey=28) |
|  | #15 | #9 OR #10 | [3 515 265](https://www.ncbi.nlm.nih.gov/pubmed/?cmd=HistorySearch&querykey=30) |
|  | #16 | #11 AND #12 AND #13 AND #14 AND #15 | 742 |
| **Embase** |  |  |  |
|  | #1 | ‘Mother’/de OR ‘Pregnancy’/de | 676 253 |
|  | #2 | Prenatal[Title/Abstract] OR Perinatal[Title/Abstract] OR Maternal[Title/Abstract] OR Gestational[Title/Abstract] OR Pre-pregnancy[Title/Abstract] OR Pregnan*[Title/Abstract] | 673 598 |
|  | #3 | ‘Body Weight’/de OR ‘Body mass’/de OR ‘Obesity’/de | 796 142 |
|  | #4 | Weight[Title/Abstract] OR body mass index[Title/Abstract] OR BMI[Title/Abstract] OR Obes*[Title/Abstract] OR Adipos*[Title/Abstract] OR overweight[Title/Abstract] | 987 375 |
|  | #5 | ‘Child’/de OR ‘Progeny’/de | 1 987 831 |
|  | #6 | Child*[Title/Abstract] OR Offspring[Title/Abstract] | 1 455 759 |
|  | #7 | ‘Attention deficit disorder’/de | 50 993 |
|  | #8 | ADHD [Title/Abstract] OR ADDH [Title/Abstract] OR ADD [Title/Abstract] OR Attention [Title/Abstract] OR Inattention [Title/Abstract] OR Hyperactiv* [Title/Abstract] OR Impulsiv* [Title/Abstract] OR Hyperkinetic disorder [Title/Abstract] | [439 689](https://www.embase.com/) |
|  | #9 | ‘Epidemiology’/de OR ‘Epidemiological data’/de OR ‘Case control study’/de OR ‘Cohort analysis’/de OR ‘Observational study’/de OR ‘Population research’/de OR ‘Longitudinal study’/de OR ‘Prospective study’/de OR ‘Follow up’/de OR ‘Retrospective study’/de | [2 802 802](https://www.embase.com/) |
|  | #10 | Case-Control Stud* [Title/Abstract] OR Cohort Stud* [Title/Abstract] OR Epidemiologic Stud* [Title/Abstract] OR  Case-control [Title/Abstract] OR Cohort[Title/Abstract] OR Epidemiolog*[Title/Abstract] OR Observational[Title/Abstract] OR Population[Title/Abstract] OR Longitudinal [Title/Abstract] OR Prospective[Title/Abstract] OR Follow-up [Title/Abstract] OR Retrospective[Title/Abstract] | 3 965 278 |
|  | #11 | #1 OR #2 | 926 330 |
|  | #12 | #3 OR #4 | 1 202 963 |
|  | #13 | #5 OR #6 | 2 366 013 |
|  | #14 | #7 OR #8 | 452 702 |
|  | #15 | #9 OR #10 | 4 721 359 |
|  | #16 | #11 AND #12 AND #13 AND #14 AND #15 | 1028 |
| **PsycINFO** |  |  |  |
|  | #1 | DE “Mothers” OR DE “Pregnancy” | 67 946 |
|  | #2 | Prenatal[Title] OR Perinatal[Title] OR Maternal[Title] OR Gestational[Title] OR Pre-pregnancy[Title] OR Pregnan*[Title] OR Women[Title] | 110 973 |
|  | #3 | Prenatal[Abstract] OR Perinatal[Abstract] OR Maternal[Abstract] OR Gestational[Abstract] OR Pre-pregnancy[Abstract] OR Pregnan*[ Abstract] OR Women[Abstract] | 311 753 |
|  | #4 | DE “Body Weight” OR DE “body mass index” OR DE “obesity” OR DE “overweight” | 41 985 |
|  | #5 | Weight[Title]OR body mass index[Title]OR BMI[Title]OR Obes*[Title]OR Adipos*[Title]OR overweight[Title] | 28 603 |
|  | #6 | Weight[Abstract] OR body mass index[Abstract] OR BMI[Abstract] OR Obes* [Abstract] OR Adipos* [Abstract] OR overweight[Abstract] | 88 040 |
|  | #7 | DE “Offspring” | 4 813 |
|  | #8 | child*[Title]OR offspring[Title] | 293 078 |
|  | #9 | child*[ Abstract]OR offspring[Abstract] | 565 546 |
|  | #10 | DE “Attention Deficit Disorder” OR DE “Attention Deficit Disorder with Hyperactivity” | 27 844 |
|  | #11 | ADHD [Title]OR ADDH [Title]OR ADD [Title]OR Attention [Title]OR Inattention [Title]OR Hyperactiv* [Title]OR Impulsiv* [Title] OR Hyperkinetic disorder [Title] | 44 761 |
|  | #12 | ADHD [Abstract]OR ADDH [Abstract]OR ADD [Abstract]OR Attention [Abstract]OR Inattention [Abstract]OR Hyperactiv* [Abstract]OR Impulsiv* [Abstract] OR Hyperkinetic disorder [Abstract] | 269 273 |
|  | #13 | DE “Cohort analysis” OR DE “Epidemiology” OR DE “Longitudinal studies” OR DE “Prospective studies” OR  DE “Follow up studies” OR DE “Retrospective studies” | 149 030 |
|  | #14 | Case-Control Stud* [Title] OR Cohort Stud* [Title] OR Epidemiologic Stud* [Title] OR Case-control [Title] OR Cohort[Title] OR Epidemiolog*[Title] OR Observational[Title] OR Population Stud* [Title] OR Longitudinal [Title] OR Prospective[Title] OR Follow up [Title] OR Retrospective[Title] | 79 165 |
|  | #15 | Case-Control Stud* [Abstract] OR Cohort Stud* [Abstract] OR Epidemiologic Stud* [Abstract] OR Case-control [Abstract] OR Cohort[Abstract] OR Epidemiolog*[ Abstract] OR Observational[Abstract] OR Population Stud*[ Abstract] OR Longitudinal [Abstract] OR Prospective[Abstract] OR Follow-up [Abstract] OR Retrospective[Abstract] | 451 760 |
|  | #16 | #1 OR #2 OR #3 | 347 477 |
|  | #17 | #4 OR #5 OR #6 | 94 096 |
|  | #18 | #7 OR #8 OR #9 | 607 150 |
|  | #19 | #10 OR #11 OR #12 | 276 668 |
|  | #20 | #13 OR #14 OR #15 | 526 721 |
|  | #21 | #16 AND #17 AND #18 AND #19 AND #20 | 242 |

| Covariates | Normal weight | Overweight/obesity | *P* |
| --- | --- | --- | --- |
| Offspring sex |  |  |  |
| Male | 326 934(51.16) | 156 151(51.19) | 0.78 |
| Female | 312 143(48.84) | 148 904(48.81) |  |
| Birth order |  |  |  |
| 1 | 284 090( 44.45) | 111 145(36.43) | <0.01 |
| 2 | 235 495( 36.85) | 113 242(37.12) |  |
| 3 | 87 971( 13.77) | 51 616(16.92) |  |
| 4+ | 31 521(4.93) | 29 052(9.52) |  |
| Offspring year of birth |  |  | <0.01 |
| 1992-95 | 229 549( 35.92) | 89 080(29.20) |  |
| 1996-99 | 178 111( 27.87) | 87 395( 28.65) |  |
| 2000-04 | 231 417( 36.21) | 128 580( 42.15) |  |
| Mother's country of birth |  |  | <0.01 |
| Sweden | 544 967( 85.27) | 253 913( 83.24) |  |
| Denmark, Finland, Iceland or Norway | 14 903( 2.33) | 7 914( 2.59) |  |
| Other | 79 207( 12.39) | 43 228( 14.17) |  |
| Maternal education |  |  | <0.01 |
| ≤9 years | 46 405( 7.41) | 31 671( 10.61) |  |
| 10-12 years | 284 514( 45.42) | 158 128( 53.00) |  |
| Postgraduate education | 295 434( 47.17) | 108 581(36.39) |  |
| Maternal age at delivery |  |  | <0.01 |
| ≤19 | 13 042( 2.04) | 4 527(1.48) |  |
| 20-24 | 102 518( 16.04) | 46 962(15.39) |  |
| 25-29 | 232 312( 36.35) | 105 431(34.56) |  |
| 30-34 | 200 992( 31.45) | 95 401(31.27) |  |
| ≥35 | 90 213(14.12) | 52 734(17.29) |  |
| Smoking during pregnancy |  |  | <0.01 |
| No | 541 596( 86.40) | 250 862( 83.90) |  |
| 1-9 cigarettes per day | 57 593( 9.19) | 30 875( 10.33) |  |
| ≥10 cigarettes per day | 27 666( 4.41) | 17 281( 5.78) |  |
| Cohabitation with child’s father at childbirth |  |  | <0.01 |
| Yes | 588 212(95.23) | 281 316(94,93) |  |
| No | 29447(4.77) | 15 032(5.07) |  |

**Table S2** The distribution of demographic characteristics of participants and maternal pre-pregnancy weight status

*Missing values: In the entire cohort, 20 217 individuals missed data for maternal highest education, 18 886 for smoking during pregnancy, 31 224 for cohabitation status; In sibling samples, 7 886 individuals missed data for maternal highest education. In cousin samples, 1 993 individuals missed data for maternal highest education, 3 065for smoking during pregnancy, 5 906 for cohabitation status.*

**Table S3** Hazard ratios for ADHD among offspring exposed to different levels of maternal pre-pregnancy BMI in cousins and sibling comparisons

| Exposure | First-born full cousins  HR(95%CI) | | | Full siblings  HR(95%CI) | | |
| --- | --- | --- | --- | --- | --- | --- |
|  | Model 1 | Model 2 | Model 3 | Model 1 | Model 2 | Model 3 |
| Pre-pregnancy normal weight | Reference | Reference | Reference | Reference | Reference | Reference |
| Pre-pregnancy overweight | 1.24(1.17-1.31) | 1.14 (1.07-1.21) | 1.10(0.98-1.23) | 1.37 (1.32-1.41) | 1.25(1.21-1.30) | 1.00 (0.91-1.11) |
| Pre-pregnancy obesity | 1.91(1.77-2.07) | 1.59 (1.46-1.72) | 1.44(1.22-1.70) | 2.09 (2.00-2.19) | 1.74(1.66-1.81) | 1.04 (0.88-1.24) |
| Obesity Class I | 1.82(1.67-1.66) | 1.54(1.41-1.69) | 1.38(1.15-1.65) | 1.98 (1.89-2.08) | 1.25(1.21-1.30) | 1.05 (0.88-1.24) |
| Obesity Class II | 2.19(1.86-2.56) | 1.71(1.45-2.00) | 1.49(1.08-2,05) | 2.44 (2.23-1.67) | 1.95(1.78-2.12) | 0.97 (0.73-1.28) |
| Obesity Class III | 2.89(1.96-4.25) | 1.96(1.29-2.98) | 1.41(0.53-3.75) | 3.35 (2.68-4.18) | 2.56(2.04-3.19) | 1.73 (0.94-3.16) |
| *P*-value for trend ^a^ | <0.00 | <0.00 | <0.00 | <0.00 | <0.00 | 0.39 |

*Model 1: Unadjusted model, using all individuals as unrelated population.*

*Model 2: Same as Model 1, using all individuals as unrelated population, but adjusted for measured confounding factors (offspring sex, birth order, year of birth, mother’s country of birth, highest maternal education, maternal age at delivery, smoking during pregnancy and cohabitation with child’s father at childbirth in first-born full cousins, and offspring sex, birth order, year of birth, maternal age at delivery, smoking during pregnancy and cohabitation with child’s father at childbirth in full siblings).*

*Model 3: Adjusted for all measured confounding factors in Model 2, and shared familial confounding within first-born cousins/full siblings.*

^a^ *P-value for trend was tested among groups: normal weight, overweight, obesity I, obesity II, obesity III.*

*ADHD: attention-deficit/hyperactivity disorder, HR: hazard ratio, CI: confidence interval.*

|  | First-born offspring | | Second-born offspring | |
| --- | --- | --- | --- | --- |
|  | Adjusted HR  (95%CI)^a^ | *P* | Adjusted HR  (95%CI)^b^ | *P* |
| Pre-pregnancy normal weight | Reference |  | Reference |  |
| Pre-pregnancy overweight | 1.29(1.22-1.37) | 0.00 | 1.27(1.20-1.34) | 0.00 |
| Pre-pregnancy obesity | 1.66(1.52-1.81) | 0.00 | 1.78(1.66-1.91) | 0.00 |
|  |  |  |  |  |
| Obesity Class I | 1.62(1.47-1.78) | 0.00 | 1.71(1.59-1.85) | 0.00 |
| Obesity Class II | 1.75(1.43-2.14) | 0.00 | 1.95(1.70-2.23) | 0.00 |
| Obesity Class III | 3.13(1.82-5.39) | 0.00 | 2.78(2.00-3.86) | 0.00 |
| *P*-value for trend *^c^* | <0.0001 |  | <0.0001 |  |

**Table S4** Stratified hazard ratios for ADHD among first- and second-born offspring exposed to different levels of maternal pre-pregnancy BMI

*^a^ N=171 508. Adjusted for offspring sex, year of birth, maternal age at delivery, smoking during pregnancy and cohabitation with child’s father at childbirth.*

*^b^ N=195 983. Adjusted for offspring sex, year of birth, maternal age at delivery, smoking during pregnancy and cohabitation with child’s father at childbirth.*

*^c^ P-value for trend was tested among groups: normal weight, overweight, obesity I, obesity II, obesity III.*

*ADHD: attention-deficit/hyperactivity disorder, HR: hazard ratio, CI: confidence interval.*

**Table S5** Hazard ratios for ADHD among offspring exposed to different levels of maternal pre-pregnancy BMI, after excluding women who ever had bariatric surgeries

| Exposure | Entire population  HR(95%CI) | | | | First-born full cousins  HR(95%CI) | | Sibling pairs  HR(95%CI) | |
| --- | --- | --- | --- | --- | --- | --- | --- | --- |
|  | Crude | *P* | Adjusted ^a^ | *P* | Adjusted ^b^ | *P* | Adjusted ^c^ | *P* |
| Pre-pregnancy normal weight | Reference |  | Reference |  | Reference |  |  |  |
| Pre-pregnancy overweight | 1.29 (1.26-1.32) | 0.00 | 1.20 (1.17-1.23) | 0.00 | 1.06 (0.95-1.19) | 0.31 | 1.00 (0.91-1.10) | 0.99 |
| Pre-pregnancy obesity | 1.78 (1.72-1.83) | 0.00 | 1.48 (1.44-1.54) | 0.00 | 1.35 (1.12-1.62) | 0.00 | 1.06 (0.88-1.24) | 0.61 |
|  |  |  |  |  |  |  |  |  |
| Obesity Class I | 1.71 (1.65-1.77) | 0.00 | 1.45 (1.40-1.51) | 0.00 | 1.35 (1.11-1.64) | 0.00 | 1.04 (0.88-1.25) | 0.59 |
| Obesity Class II | 2.01 (1.87-2.15) | 0.00 | 1.58 (1.47-1.70) | 0.00 | 1.17 (0.78-1.72) | 0.46 | 0.97 (0.73-1.30) | 0.95 |
| Obesity Class III | 2.64 (2.19-3.16) | 0.00 | 2.02 (1.67-2.45) | 0.00 | 1.33 (0.38-4.71) | 0.65 | 1.73 (0.93-3.21) | 0.08 |
| P-value for trend ^d^ | <0.0001 |  | <0.0001 |  | <0.0001 |  | 0.39 |  |

*^a^ N=865 793. Adjusted for offspring sex, birth order, year of birth, mother’s country of birth, highest maternal education, maternal age at delivery, smoking during pregnancy and cohabitation with child’s father at childbirth.*

*^b^ N=143 209. Adjusted for offspring sex, birth order, year of birth, mother’s country of birth, highest maternal education, maternal age at delivery, smoking during pregnancy and cohabitation with child’s father at childbirth, and shared familial confounding within first-born cousin.*

*^c^ N=431 690. Adjusted for offspring sex, birth order, year of birth, maternal age at delivery, smoking during pregnancy and cohabitation with child’s father at childbirth, and shared familial confounding within first-second born sibling pairs.*

*^d^ P-value for trend was tested among groups: normal weight, overweight, obesity I, obesity II, obesity III.*

*ADHD: attention-deficit/hyperactivity disorder, HR: hazard ratio, CI: confidence interval.*

**Table S6** Sample sizes of discordant cousin- and sibling-pairs

|  | Cousin pairs | | Sibling pairs | |
| --- | --- | --- | --- | --- |
|  | Discordant for exposure | Also discordant for ADHD | Discordant for exposure | Also discordant for ADHD |
| Pre-pregnancy normal weight | Reference |  | Reference |  |
| Pre-pregnancy overweight | 41515 | 3478 | 30724 | 2130 |
| Obesity Class I | 10181 | 1061 | 1083 | 91 |
| Obesity Class II | 2311 | 267 | 90 | 7 |
| Obesity Class III | 304 | 36 | 9 | 1 |

*ADHD: attention-deficit/hyperactivity disorder.*
